# 2,2,2-Trifluoro-*N*-(isoquinolin-5-ylmeth­yl)acetamide

**DOI:** 10.1107/S1600536809052994

**Published:** 2009-12-12

**Authors:** Alan R. Kennedy, Abedawn I. Khalaf, Colin J. Suckling

**Affiliations:** a Department of Pure & Applied Chemistry, University of Strathclyde, 295 Cathedral Street, Glasgow G1 1XL, Scotland

## Abstract

The mol­ecular structure of the title compound at 123 K, C_12_H_9_F_3_N_2_O, presents a rotationally disordered CF_3_ group. Hydrogen bonds between the amide NH group and the N atom of the isoquinoline form a chain in the *b*-axis direction. The packed structure forms alternate layers of isoquinoline and amide groups parallel to the *ab* plane.

## Related literature

In the search for biologically active compounds in the area of anti-inflammatory and pain relief drugs, we have found a class of compounds that act as potent antagonists or agonists of the vanilloid VR1 receptor. These have been shown to be useful in the treatment and prevention of inflammatory and other pain conditions in mammals, see: Jetter *et al.* (2007 ▶, 2008 ▶); Codd *et al.* (2003 ▶). The title compound was prepared as a precursor for more complex compounds. For analysis of the structures of analogous naphthalenes, see: Weinstein & Leiserowitz (1980 ▶). For a discussion on disorder in crystal structures, see: Müller (2009 ▶).
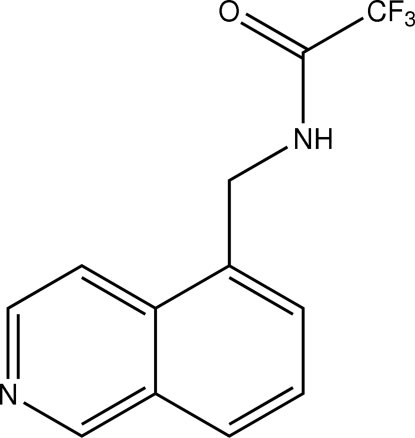


## Experimental

### 

#### Crystal data

C_12_H_9_F_3_N_2_O
*M* *_r_* = 254.21
Monoclinic, 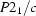

*a* = 7.2308 (7) Å
*b* = 8.3498 (11) Å
*c* = 18.157 (2) Å
β = 90.583 (9)°
*V* = 1096.2 (2) Å^3^
*Z* = 4
Mo *K*α radiation
μ = 0.14 mm^−1^
*T* = 123 K
0.45 × 0.12 × 0.02 mm

#### Data collection

Oxford Diffraction Xcalibur S diffractometer
Absorption correction: multi-scan (*CrysAlis RED*; Oxford Diffraction, 2007 ▶) *T*_min_ = 0.717, *T*_max_ = 1.000
4377 measured reflections
2071 independent reflections
1346 reflections with *I* > 2σ(*I*)
*R*_int_ = 0.031

#### Refinement

*R*[*F* ^2^ > 2σ(*F* ^2^)] = 0.051
*wR*(*F* ^2^) = 0.133
*S* = 1.06
2071 reflections
191 parameters
111 restraints
H atoms treated by a mixture of independent and constrained refinement
Δρ_max_ = 0.30 e Å^−3^
Δρ_min_ = −0.45 e Å^−3^

### 

Data collection: *CrysAlis CCD* (Oxford Diffraction, 2007 ▶); cell refinement: *CrysAlis CCD*; data reduction: *CrysAlis RED* (Oxford Diffraction, 2007 ▶); program(s) used to solve structure: *SHELXS97* (Sheldrick, 2008 ▶); program(s) used to refine structure: *SHELXL97* (Sheldrick, 2008 ▶); molecular graphics: *ORTEP-3* (Farrugia, 1997 ▶); software used to prepare material for publication: *SHELXL97*.

## Supplementary Material

Crystal structure: contains datablocks global, I. DOI: 10.1107/S1600536809052994/tk2599sup1.cif

Structure factors: contains datablocks I. DOI: 10.1107/S1600536809052994/tk2599Isup2.hkl

Additional supplementary materials:  crystallographic information; 3D view; checkCIF report

## Figures and Tables

**Table 1:** Hydrogen-bond geometry (Å, °)

*D*—H⋯*A*	*D*—H	H⋯*A*	*D*⋯*A*	*D*—H⋯*A*
N1—H1*N*⋯N2^i^	0.84 (3)	2.05 (3)	2.847 (3)	158 (3)

Symmetry code: (i) 
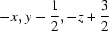
.

**Fig. 1.**
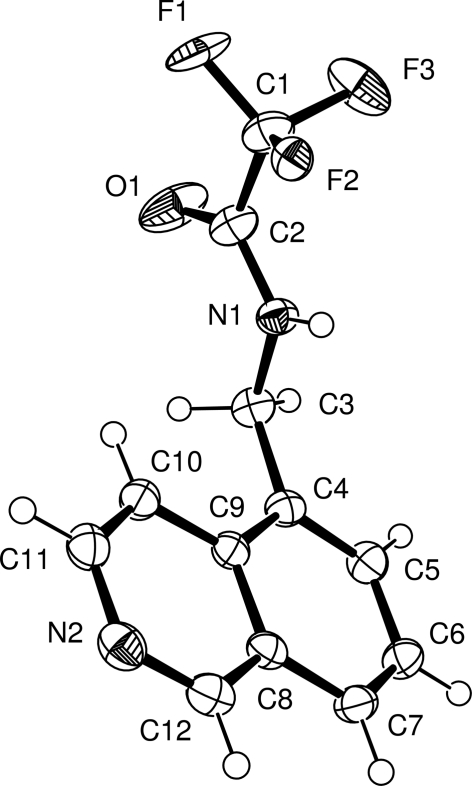
The molecular structure and atomic labelling, showing 40% probability displacement ellipsoids. H atoms are shown as spheres of arbitrary radius. Minor disorder components are not shown.

**Fig. 2.**
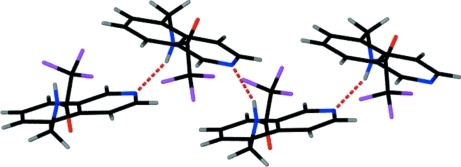
Part of the hydrogen bonded chains formed along the b direction. O atoms are red, N atoms blue, F atoms pink and C-atoms are black. Hydrogen bonds are shown as dashed lines.

**Fig. 3.**
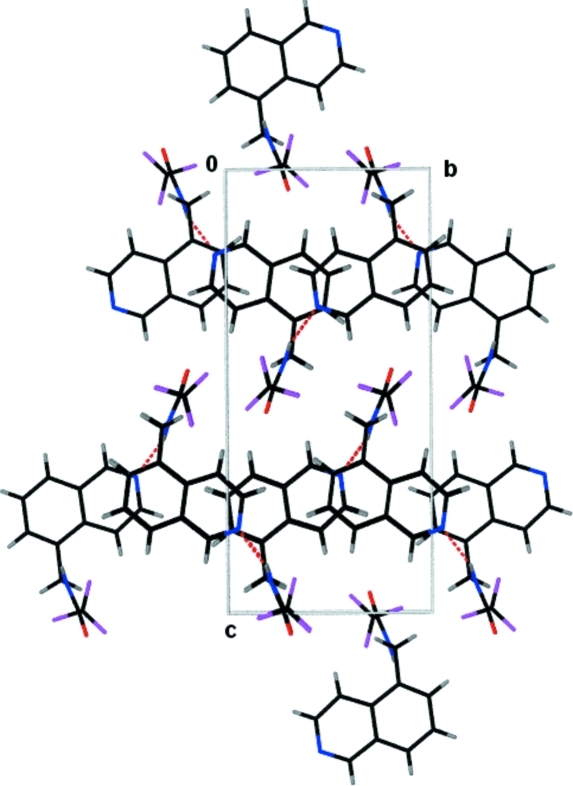
Packed structure viewed down the a-axis.

## supplementary crystallographic information

### Comment

In the search for novel biologically active compounds in the area of anti-inflammatory and pain relief drugs, we have found a class of compounds acting as potent antagonists or agonists of the vanilloid VR1 receptor. These have been shown to be useful in the treatment and prevention of inflammatory and other pain conditions in mammals (Jetter *et al.*, 2007; Jetter *et al.*, 2008; Codd *et al.*, 2003). The title compound was prepared as an important precursor for more complex compounds. Surprisingly, reaction of n-hydroxymethyl trifluoroacetamide with isoquinoline in the presence of sulfuric acid afforded a single positional isomer after purification by column chromatography.

The plane of the quinolene ring forms a dihedral angle of 76.2 (2) ° with the amide plane, see Fig. 1. Similar conformations were found in a study of similar naphthalene derivatives - though the analogous CF_3_ compound had an angle of 89 ° (Weinstein & Leiserowitz, 1980). The CF_3_ group is disordered by rotation about the C—CF_3_ bond (Müller, 2009).

A major difference between the naphthalene derivatives and the present compound is seen in the intermolecular contacts. The naphthalenes bond through amide to amide (N—H···O) hydrogen bonds. However, in the quinolene, the presence of a hetero atom enables N—H···N bonds. These form extended chains running along the *b* direction, see Fig. 2.

The packing diagram, Fig. 3, illustrates the layered nature of this structure. Traveling along the *c* direction there are alternate quinolene layers and amide layers. The closest π interaction, connecting molecules along the *a* direction, in the quinolene layer is slightly outside the sum of van der Waals distances (C5···C12 3.486 (4) Å).

### Experimental

Isoquinoline (1.29 g, 10 mmol) in concentrated sulfuric acid (50 ml) was cooled to 293 K. n-Hydroxymethyl trifluoroacetamide (1.43 g, 10.00 mmol) was added in portions. After 15 min, the reaction mixture was allowed to warm to room temperature and stirred for 16 h. The clear light-brown reaction mixture was then poured onto 200 g of ice, then concentrated ammonium hydroxide was added dropwise until the reaction mixture was basic to pH paper. After extraction with 100 ml of dichloromethane, the organic layer was washed (2 *x* 100 ml brine), dried over MgSO_4_ and then evaporated under reduced pressure. The residue was applied to a silica gel column and eluted with 1:2 ethyl acetate:hexane [*R*_F_ = 1/5]. This gave the product as a crystalline solid (2.03 g, 80%), m.p. 435 - 438 K. IR (KBr): 1716, 1624, 1563, 1380, 1211, 1141, 1034, 832, 755, 707 cm ^-1^. _1_H NMR (DMSO-d_6_): 10.08 (1*H*, s), 9.35 (1*H*, d, J = 0.8 Hz), 8.57 (1*H*, d, J = 6.0 Hz), 8.10 (1*H*, dd, J = 6.9 & 1.9 Hz), 7.96 (1*H*, d, J = 6.0 Hz), 7.71–7.65 (2*H*, m), 4.85 (2*H*, s) ppm.

### Refinement

The F atoms of the CF_3_ group are disordered by rotation about the C2—C1 bond. After several trial calculations, a model with three separate groups of F atom positions was adopted. Site occupancy factors are 0.5 for F1 to F3, 0.3 for F4 to F6 and 0.2 for F7 to F9. Only F1 to F3 were refined anisotropically. Restraints were placed on the C—F distances (1.33 Å) and to encourage similarity in the F atom *U*_ij_ values (Müller, 2009).

The amide-H atom was found by difference synthesis and refined isotropically. All other H atoms were positioned geometrically at distances of 0.95 and 0.99 Å from the parent C atom for CH and CH_2_ groups respectively. For these atoms, a riding model was used with *U*_iso_(H) values constrained to be 1.2 times *U*_eq_ of the parent C atom.

### Crystal data

**Table:**

C_12_H_9_F_3_N_2_O	*F*(000) = 520
*M**_r_* = 254.21	*D*_x_ = 1.540 Mg m^−^^3^
Monoclinic, *P*2_1_/*c*	Melting point = 435–438 K
Hall symbol: -P 2ybc	Mo *K*α radiation, λ = 0.71073 Å
*a* = 7.2308 (7) Å	Cell parameters from 1879 reflections
*b* = 8.3498 (11) Å	θ = 2.7–30.1°
*c* = 18.157 (2) Å	µ = 0.14 mm^−^^1^
β = 90.583 (9)°	*T* = 123 K
*V* = 1096.2 (2) Å^3^	Blade, colourless
*Z* = 4	0.45 × 0.12 × 0.02 mm

### Data collection

**Table:**

Oxford Diffraction Xcalibur S diffractometer	2071 independent reflections
Radiation source: fine-focus sealed tube	1346 reflections with *I* > 2σ(*I*)
graphite	*R*_int_ = 0.031
Detector resolution: 16.0268 pixels mm^-1^	θ_max_ = 26.0°, θ_min_ = 2.7°
ω scans	*h* = −8→8
Absorption correction: multi-scan (*CrysAlis RED*; Oxford Diffraction, 2007)	*k* = −10→9
*T*_min_ = 0.717, *T*_max_ = 1.000	*l* = −21→22
4377 measured reflections	

### Refinement

**Table:**

Refinement on *F*^2^	Primary atom site location: structure-invariant direct methods
Least-squares matrix: full	Secondary atom site location: difference Fourier map
*R*[*F*^2^ > 2σ(*F*^2^)] = 0.051	Hydrogen site location: inferred from neighbouring sites
*wR*(*F*^2^) = 0.133	H atoms treated by a mixture of independent and constrained refinement
*S* = 1.06	*w* = 1/[σ^2^(*F*_o_^2^) + (0.0703*P*)^2^ + 0.0571*P*] where *P* = (*F*_o_^2^ + 2*F*_c_^2^)/3
2071 reflections	(Δ/σ)_max_ < 0.001
191 parameters	Δρ_max_ = 0.30 e Å^−^^3^
111 restraints	Δρ_min_ = −0.45 e Å^−^^3^

### Special details

**Table:**

Geometry. All e.s.d.'s (except the e.s.d. in the dihedral angle between two l.s. planes) are estimated using the full covariance matrix. The cell e.s.d.'s are taken into account individually in the estimation of e.s.d.'s in distances, angles and torsion angles; correlations between e.s.d.'s in cell parameters are only used when they are defined by crystal symmetry. An approximate (isotropic) treatment of cell e.s.d.'s is used for estimating e.s.d.'s involving l.s. planes.
Refinement. Refinement of *F*^2^ against ALL reflections. The weighted *R*-factor *wR* and goodness of fit *S* are based on *F*^2^, conventional *R*-factors *R* are based on *F*, with *F* set to zero for negative *F*^2^. The threshold expression of *F*^2^ > σ(*F*^2^) is used only for calculating *R*-factors(gt) *etc*. and is not relevant to the choice of reflections for refinement. *R*-factors based on *F*^2^ are statistically about twice as large as those based on *F*, and *R*- factors based on ALL data will be even larger.

### Fractional atomic coordinates and isotropic or equivalent isotropic displacement parameters (Å^2^)

**Table:**

	*x*	*y*	*z*	*U*_iso_*/*U*_eq_	Occ. (<1)
F1	−0.3651 (5)	0.3984 (6)	1.0372 (2)	0.0468 (10)	0.50
F2	−0.3741 (5)	0.3253 (6)	0.92452 (18)	0.0266 (10)	0.50
F3	−0.3909 (5)	0.1505 (5)	1.0096 (3)	0.0490 (10)	0.50
F4	−0.3837 (11)	0.2632 (9)	0.9261 (3)	0.042 (2)*	0.30
F5	−0.3858 (10)	0.2219 (12)	1.0445 (4)	0.0590 (19)*	0.30
F6	−0.3263 (9)	0.4512 (6)	1.0002 (4)	0.0530 (17)*	0.30
F7	−0.3639 (12)	0.3146 (15)	1.0568 (3)	0.031 (2)*	0.20
F8	−0.3620 (17)	0.3826 (12)	0.9363 (5)	0.044 (3)*	0.20
F9	−0.3770 (13)	0.1449 (9)	0.9730 (6)	0.043 (2)*	0.20
O1	−0.0083 (3)	0.3065 (3)	1.04575 (11)	0.0574 (7)	
N1	−0.0214 (3)	0.2113 (3)	0.92904 (12)	0.0269 (5)	
N2	0.2036 (3)	0.5534 (3)	0.69021 (12)	0.0323 (6)	
C1	−0.3025 (3)	0.2895 (3)	0.98984 (12)	0.0345 (7)	
C2	−0.0925 (4)	0.2699 (3)	0.99042 (14)	0.0323 (7)	
C3	0.1779 (3)	0.1840 (3)	0.92121 (13)	0.0291 (6)	
H3A	0.2135	0.0859	0.9485	0.035*	
H3B	0.2465	0.2752	0.9431	0.035*	
C4	0.2312 (3)	0.1660 (3)	0.84124 (13)	0.0248 (6)	
C5	0.2834 (3)	0.0198 (3)	0.81357 (14)	0.0285 (6)	
H5	0.2895	−0.0697	0.8458	0.034*	
C6	0.3287 (3)	−0.0013 (4)	0.73828 (13)	0.0297 (6)	
H6	0.3651	−0.1039	0.7211	0.036*	
C7	0.3203 (3)	0.1237 (3)	0.69074 (13)	0.0294 (6)	
H7	0.3497	0.1090	0.6403	0.035*	
C8	0.2672 (3)	0.2763 (3)	0.71693 (13)	0.0249 (6)	
C9	0.2235 (3)	0.2993 (3)	0.79266 (13)	0.0230 (6)	
C10	0.1718 (3)	0.4552 (3)	0.81438 (13)	0.0264 (6)	
H10	0.1419	0.4768	0.8642	0.032*	
C11	0.1651 (3)	0.5738 (3)	0.76334 (14)	0.0305 (6)	
H11	0.1314	0.6780	0.7794	0.037*	
C12	0.2528 (3)	0.4093 (4)	0.66921 (14)	0.0306 (6)	
H12	0.2805	0.3935	0.6187	0.037*	
H1N	−0.089 (4)	0.189 (4)	0.8928 (16)	0.038 (8)*	

### Atomic displacement parameters (Å^2^)

**Table:**

	*U*^11^	*U*^22^	*U*^33^	*U*^12^	*U*^13^	*U*^23^
F1	0.0389 (18)	0.068 (3)	0.0337 (18)	0.0250 (19)	0.0054 (15)	−0.022 (2)
F2	0.0278 (17)	0.020 (2)	0.0314 (19)	0.0033 (19)	0.0001 (12)	0.0036 (17)
F3	0.0405 (18)	0.048 (2)	0.058 (2)	−0.0050 (16)	0.0009 (18)	0.038 (2)
O1	0.0464 (13)	0.094 (2)	0.0313 (11)	0.0251 (12)	−0.0109 (9)	−0.0248 (12)
N1	0.0266 (11)	0.0317 (14)	0.0223 (11)	0.0007 (10)	0.0021 (9)	−0.0017 (11)
N2	0.0275 (11)	0.0318 (16)	0.0376 (13)	−0.0004 (10)	0.0000 (9)	0.0053 (12)
C1	0.0418 (16)	0.0372 (19)	0.0247 (14)	0.0078 (14)	0.0052 (11)	0.0029 (13)
C2	0.0382 (15)	0.0337 (18)	0.0250 (14)	0.0089 (13)	0.0028 (11)	0.0004 (13)
C3	0.0300 (13)	0.0301 (17)	0.0271 (13)	0.0042 (12)	0.0006 (10)	0.0016 (12)
C4	0.0197 (12)	0.0290 (17)	0.0258 (13)	0.0025 (11)	−0.0001 (9)	−0.0002 (12)
C5	0.0279 (13)	0.0236 (16)	0.0340 (14)	0.0044 (11)	0.0000 (10)	0.0032 (13)
C6	0.0286 (14)	0.0272 (16)	0.0335 (14)	0.0035 (11)	0.0035 (10)	−0.0048 (13)
C7	0.0263 (13)	0.0356 (18)	0.0263 (13)	0.0000 (12)	0.0042 (10)	−0.0055 (13)
C8	0.0203 (12)	0.0283 (15)	0.0262 (13)	−0.0023 (11)	0.0014 (9)	0.0011 (12)
C9	0.0182 (11)	0.0253 (15)	0.0255 (13)	−0.0007 (10)	−0.0007 (9)	−0.0004 (12)
C10	0.0235 (13)	0.0270 (16)	0.0286 (13)	0.0004 (11)	−0.0003 (10)	−0.0024 (12)
C11	0.0246 (13)	0.0273 (17)	0.0396 (15)	0.0002 (11)	0.0001 (10)	−0.0013 (13)
C12	0.0264 (13)	0.0371 (18)	0.0284 (13)	−0.0021 (12)	0.0013 (10)	0.0029 (13)

### Geometric parameters (Å, °)

**Table:**

F1—C1	1.334 (3)	C3—H3A	0.9900
F2—C1	1.324 (4)	C3—H3B	0.9900
F3—C1	1.374 (4)	C4—C5	1.374 (4)
F4—C1	1.311 (5)	C4—C9	1.421 (4)
F5—C1	1.295 (5)	C5—C6	1.420 (3)
F6—C1	1.375 (5)	C5—H5	0.9500
F7—C1	1.316 (5)	C6—C7	1.356 (4)
F8—C1	1.314 (5)	C6—H6	0.9500
F9—C1	1.356 (5)	C7—C8	1.414 (4)
O1—C2	1.209 (3)	C7—H7	0.9500
N1—C2	1.326 (3)	C8—C12	1.412 (4)
N1—C3	1.467 (3)	C8—C9	1.427 (3)
N1—H1N	0.84 (3)	C9—C10	1.411 (4)
N2—C12	1.312 (3)	C10—C11	1.357 (4)
N2—C11	1.370 (3)	C10—H10	0.9500
C1—C2	1.527 (4)	C11—H11	0.9500
C3—C4	1.514 (3)	C12—H12	0.9500
			
C2—N1—C3	121.9 (2)	C4—C3—H3B	109.3
C2—N1—H1N	121 (2)	H3A—C3—H3B	108.0
C3—N1—H1N	117.2 (19)	C5—C4—C9	118.5 (2)
C12—N2—C11	117.0 (2)	C5—C4—C3	120.8 (2)
F5—C1—F4	113.3 (6)	C9—C4—C3	120.6 (2)
F8—C1—F7	118.6 (8)	C4—C5—C6	122.0 (2)
F2—C1—F1	106.9 (3)	C4—C5—H5	119.0
F8—C1—F9	103.5 (7)	C6—C5—H5	119.0
F7—C1—F9	102.3 (7)	C7—C6—C5	120.5 (3)
F2—C1—F3	104.2 (3)	C7—C6—H6	119.7
F1—C1—F3	104.2 (3)	C5—C6—H6	119.7
F5—C1—F6	105.3 (5)	C6—C7—C8	119.4 (2)
F4—C1—F6	103.3 (5)	C6—C7—H7	120.3
F5—C1—C2	114.7 (4)	C8—C7—H7	120.3
F4—C1—C2	115.1 (4)	C12—C8—C7	121.4 (2)
F8—C1—C2	112.7 (6)	C12—C8—C9	118.0 (2)
F7—C1—C2	110.8 (4)	C7—C8—C9	120.6 (2)
F2—C1—C2	114.2 (2)	C10—C9—C4	123.9 (2)
F1—C1—C2	114.4 (2)	C10—C9—C8	117.1 (2)
F9—C1—C2	107.4 (5)	C4—C9—C8	119.0 (2)
F3—C1—C2	111.9 (3)	C11—C10—C9	119.3 (2)
F6—C1—C2	103.3 (3)	C11—C10—H10	120.3
O1—C2—N1	126.5 (3)	C9—C10—H10	120.3
O1—C2—C1	118.1 (2)	C10—C11—N2	124.4 (3)
N1—C2—C1	115.3 (2)	C10—C11—H11	117.8
N1—C3—C4	111.6 (2)	N2—C11—H11	117.8
N1—C3—H3A	109.3	N2—C12—C8	124.2 (2)
C4—C3—H3A	109.3	N2—C12—H12	117.9
N1—C3—H3B	109.3	C8—C12—H12	117.9
			
C3—N1—C2—O1	−0.3 (5)	N1—C3—C4—C9	69.5 (3)
C3—N1—C2—C1	−179.4 (2)	C9—C4—C5—C6	−0.3 (4)
F5—C1—C2—O1	−52.4 (6)	C3—C4—C5—C6	178.1 (2)
F4—C1—C2—O1	173.4 (5)	C4—C5—C6—C7	−0.4 (4)
F8—C1—C2—O1	122.9 (6)	C5—C6—C7—C8	0.5 (4)
F7—C1—C2—O1	−12.8 (7)	C6—C7—C8—C12	−178.7 (2)
F2—C1—C2—O1	148.2 (4)	C6—C7—C8—C9	0.3 (3)
F1—C1—C2—O1	24.5 (5)	C5—C4—C9—C10	179.9 (2)
F9—C1—C2—O1	−123.8 (5)	C3—C4—C9—C10	1.5 (4)
F3—C1—C2—O1	−93.7 (4)	C5—C4—C9—C8	1.0 (3)
F6—C1—C2—O1	61.6 (4)	C3—C4—C9—C8	−177.4 (2)
F5—C1—C2—N1	126.8 (6)	C12—C8—C9—C10	−1.0 (3)
F4—C1—C2—N1	−7.4 (5)	C7—C8—C9—C10	−180.0 (2)
F8—C1—C2—N1	−57.9 (7)	C12—C8—C9—C4	178.0 (2)
F7—C1—C2—N1	166.4 (6)	C7—C8—C9—C4	−1.0 (3)
F2—C1—C2—N1	−32.7 (4)	C4—C9—C10—C11	−178.6 (2)
F1—C1—C2—N1	−156.3 (4)	C8—C9—C10—C11	0.3 (3)
F9—C1—C2—N1	55.4 (5)	C9—C10—C11—N2	0.8 (4)
F3—C1—C2—N1	85.5 (4)	C12—N2—C11—C10	−1.1 (3)
F6—C1—C2—N1	−119.2 (4)	C11—N2—C12—C8	0.3 (4)
C2—N1—C3—C4	−162.6 (2)	C7—C8—C12—N2	179.7 (2)
N1—C3—C4—C5	−108.9 (3)	C9—C8—C12—N2	0.8 (4)

### Hydrogen-bond geometry (Å, °)

**Table:**

*D*—H···*A*	*D*—H	H···*A*	*D*···*A*	*D*—H···*A*
N1—H1N···N2^i^	0.84 (3)	2.05 (3)	2.847 (3)	158 (3)

Symmetry codes: (i) −*x*, *y*−1/2, −*z*+3/2.
